# Complex Network Theory Applied to the Growth of Kuala Lumpur’s Public Urban Rail Transit Network

**DOI:** 10.1371/journal.pone.0139961

**Published:** 2015-10-08

**Authors:** Rui Ding, Norsidah Ujang, Hussain bin Hamid, Jianjun Wu

**Affiliations:** 1 Faculty of Design and Architecture, Universiti Putra Malaysia, Serdang, Selangor, Malaysia; 2 Faculty of Engineering, Universiti Putra Malaysia, Serdang, Selangor, Malaysia; 3 State Key Laboratory of Rail Traffic Control and Safety, Beijing Jiaotong University, Beijing, China; Beihang University, CHINA

## Abstract

Recently, the number of studies involving complex network applications in transportation has increased steadily as scholars from various fields analyze traffic networks. Nonetheless, research on rail network growth is relatively rare. This research examines the evolution of the Public Urban Rail Transit Networks of Kuala Lumpur (PURTNoKL) based on complex network theory and covers both the topological structure of the rail system and future trends in network growth. In addition, network performance when facing different attack strategies is also assessed. Three topological network characteristics are considered: connections, clustering and centrality. In PURTNoKL, we found that the total number of nodes and edges exhibit a linear relationship and that the average degree stays within the interval [2.0488, 2.6774] with heavy-tailed distributions. The evolutionary process shows that the cumulative probability distribution (CPD) of degree and the average shortest path length show good fit with exponential distribution and normal distribution, respectively. Moreover, PURTNoKL exhibits clear cluster characteristics; most of the nodes have a 2-core value, and the CPDs of the centrality’s closeness and betweenness follow a normal distribution function and an exponential distribution, respectively. Finally, we discuss four different types of network growth styles and the line extension process, which reveal that the rail network’s growth is likely based on the nodes with the biggest lengths of the shortest path and that network protection should emphasize those nodes with the largest degrees and the highest betweenness values. This research may enhance the networkability of the rail system and better shape the future growth of public rail networks.

## Introduction

The traffic network represents the fundamental structure of a city. As the carrier of its transportation activities and functions, it can be considered the aorta of a city’s economy and its operational development. A city’s traffic network is its most crucial subsystem for ensuring that the city runs in a stable and orderly fashion. Traffic is highly connected to and influenced by all levels of society, including the relationship among surrounding population distributions, transportation needs, economic development, culture, and geological conditions and landforms [1]. All these factors result in the need for different modes of transportation. With regard to public rail transit, in particular, its convenience and energy conservation makes this system of particular interest for researchers [2].

Most traditional traffic indicators are either graph or geographical indicators [3]. Garrison was the first to analyze public transportation networks [4], and after decades of improvement, scholars now know the particular factors that influence traffic hub nodes and network coverage, and have identified evaluation methods to consider both the topology and geography of networks [2–5]. The graph category measures and evaluates topological elements such as lines and networks, node degrees, node weights, edge weights, connectivity, complexity, and loops within the network. The geographical category defines more comprehensive indicators of a real network such as the lengths of edges and the average interspaces between stations. Derrible and Kennedy also made a significant contribution to transportation network analysis by using a new methodology to redraw metro networks into graphs using two indicators: directness and structural connectivity [6]. These researchers characterized 33 metro networks around the world, reviewed the impact of network size, and discussed the implications for topology. Quintero-Cano *et al*. [7] focused on pre-existing network indicators, whereas Kansky summarized the definitions of transit network properties [3]. In addition, Quintero-Cano [8] presented several macro-level prediction models for transit infrastructure, transportation network topology, transit route design, and transit performance and operations.

Researchers focused on the definitions of networks and spatial characteristics have used complex network theory to demonstrate that urban traffic networks—including rail, roads and streets—consist of the features that characterize complex networks; these researchers have also explored previously undiscovered macroscopic properties of traffic networks, defined new statistical parameters, and extensively investigated infrastructure networks [9–12]. Latora and Marchiori [13–15] and Wu, Sun, Gao *et al*. [16–21] used the statistical properties of individual choice and cascading failures to reveal the dynamic behavior of scale-free urban traffic networks. Computing the distribution degree, average path length, and clustering coefficient of a public transportation system in Shanghai, Li *et al*. [22] assessed the complex characteristics of that system while simultaneously exploring the error tolerance and attack vulnerability of the network. Their findings indicated that the public transportation network had a short average path length and high clustering coefficient; moreover, these findings were highly robust to errors but weakly robust to targeted attacks. Zhang *et al*. [23] discovered the universal characteristics of urban rail transit networks (URTNs) from six indicators and calculated the topological efficiency of the network and the clustering coefficient of the node. After comparing the basic spatial topological characteristics of rail transit networks from approximately 30 cities worldwide, these authors found that the average node degrees of URTNs are between 2 and 2.45; in addition, most of the average shortest path lengths fell into the interval 10 to 16, and the average betweenness of the nodes and edges exhibited a positive correlation with the number of stations. Other researchers who have studied the rail transit system have analyzed its statistical characteristics and evaluated the connectivity and reliability of the network using robustness theory [24–26].

However, insufficient research has been undertaken on rail transit networks and their complex network performance during the network expansion process. Importantly, the growth of a transportation network not only involves local traffic demand but also derives from optimizing transportation capacity across the entire network. As a result, concern for the growth process of a network will yield tremendous benefits over the long run. In this research, we will assess the network growth process, illustrate the network structure, and assess the performance and the evolution of the Public Urban Rail Transit Network of Kuala Lumpur (PURTNoKL). We also present a dynamic cascade process to show how limited structural change can affect the entire network and determine which components are most valuable within the network and which have the potential for future development. This analysis thus aims to enhance PURTNoKL’s networkability with limited capital input and to ensure that future growth will be shaped practically and scientifically.

## Nature Evolution Process of PURTNoKL

In Malaysia, Mohamad studied the rail network expansion process in Kuala Lumpur based on daily service times, coverage area, automobile equipment, running policies, traffic capacity, service objects and line integration to describe the basic rail transit situation, including necessary facilities, bus services, and private cars [27]. Moreover, Mohamad assumed that the future development of the public URTNs in Kuala Lumpur would mean expanding the network by adding nodes and improving service standards.

Rapid urban population growth and increasing traffic congestion emphasize the need for additional public transport [28]. The modern rail transport system has been widely used since 1995 in Greater Kuala Lumpur and the Klang Valley [29]. The rail system consists of two primary lines that are integrated and referred to as Keretapi Tanah Melayu (KTM). The first line is the Seremban Line, which runs nearly 160 km from Rawang station to Seremban and is operated by KTM Komuter. During normal operations, 3 more stations are active on this line: Kepong Sentral, KL Sentral, and Mid Valley. After introduction of the Rawang-Tanjung Malim shuttle service in 2007, the Seremban line expanded from Seremban Station to Rembau, acting as the pipeline connecting Kuala Lumpur to the surrounding suburbs. The second line is the Port Klang Line, which was opened on August 14, 1995, and stops at 21 stations over the 45 km between the Batu Caves and Port Klang. Adding 2 stations expanded the line from Putra to the Batu Caves, which is a famous tourist spot near Kuala Lumpur.

In 1996, there were two hinged and medium-capacity rapid transit routes, the Ampang Line and the Sri Petaling Line, that measured 27 km in total length and were operated by Rapid Rail. A section between Chan Sow Lin and Sri Petaling opened later, and the Sultan Ismail and Sentul Timur stations began operations in 1998. An extension is planned for development in two stages; stage one will extend from Seri Petaling to Bandar Kinrara and is expected to begin operations in 2015, and stage two will run from IOI Puchong Jaya to Putra Heights and will open in March 2016.

The Kelana Jaya Line is also part of two medium-capacity rapid transit systems. Referred to as the LRT, it was integrated into the Ampang Line in November 2011. It consists of 24 elevated light rail stations and 5 underground stations. The first section, which runs from Subang Depot to Pasar Seni, began operations in September 1998, whereas the second section, running from Pasar Seni to Putra Terminal, began operations in June 1999. Thus, the primary network of Kuala Lumpur was formed at that time, mainly covering the downtown area [30]. An extension from Kelana Jaya station to Putra Heights is expected to commence operations in 2016 and will join with Sri Petaling Line to form a loop.

The KLIA Express has only two stations—KL Sentral and the Kuala Lumpur International Airport (KLIA)–and non-stop express service has been running on this line since 2002. The KLIA Transit line is quite different; it has 3 stops, is connected to the Bandar Tasik Selatan transfer station (which is connected to KTM and LRT, in addition to bus, and taxi services), and has also been running since 2002.

The KL Monorail Line is the only urban monorail system in inner Kuala Lumpur. It is 8.6 km long, stops at 11 stations, has been linked with KL Sentral since August 2003, and is connected to the Ampang LRT Line by 2 stations. This line runs straight through the Bukit Bintang area, which is home to the most developed shopping malls in the city.

The MRT Sungai Buloh-Kajang Line, which is expected to be completed in 2017, will link the entire network from west to east, starting from Sungai Buloh station, running across KL Sentral and the Bukit Bintang area, and connecting the Kelana Jaya, Ampang, Sri Petaling and KL Monorail Lines before terminating at Kajang station.

## Methods

In this section, we will review and introduce the indicators used in the network design of public rail systems to test and calculate the performance and status of the PURTNoKL. First, we reviewed the static characteristics of networks. The network was represented using graph theory and was analyzed using the PURTNoKL’s natural growth process. We then developed a network growth matrix with a time series.

**Fig 1:**
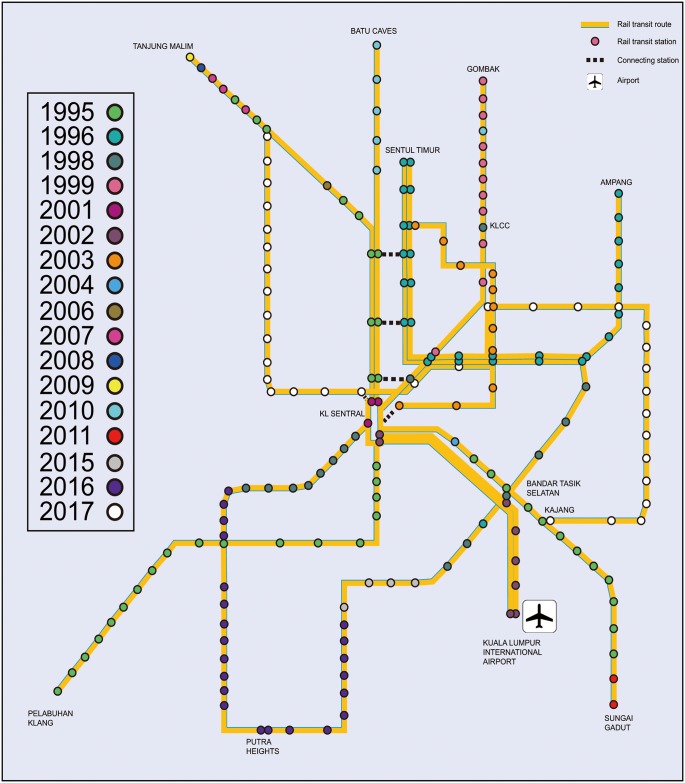
The PURTNoKL development plan.

**Fig 2:**
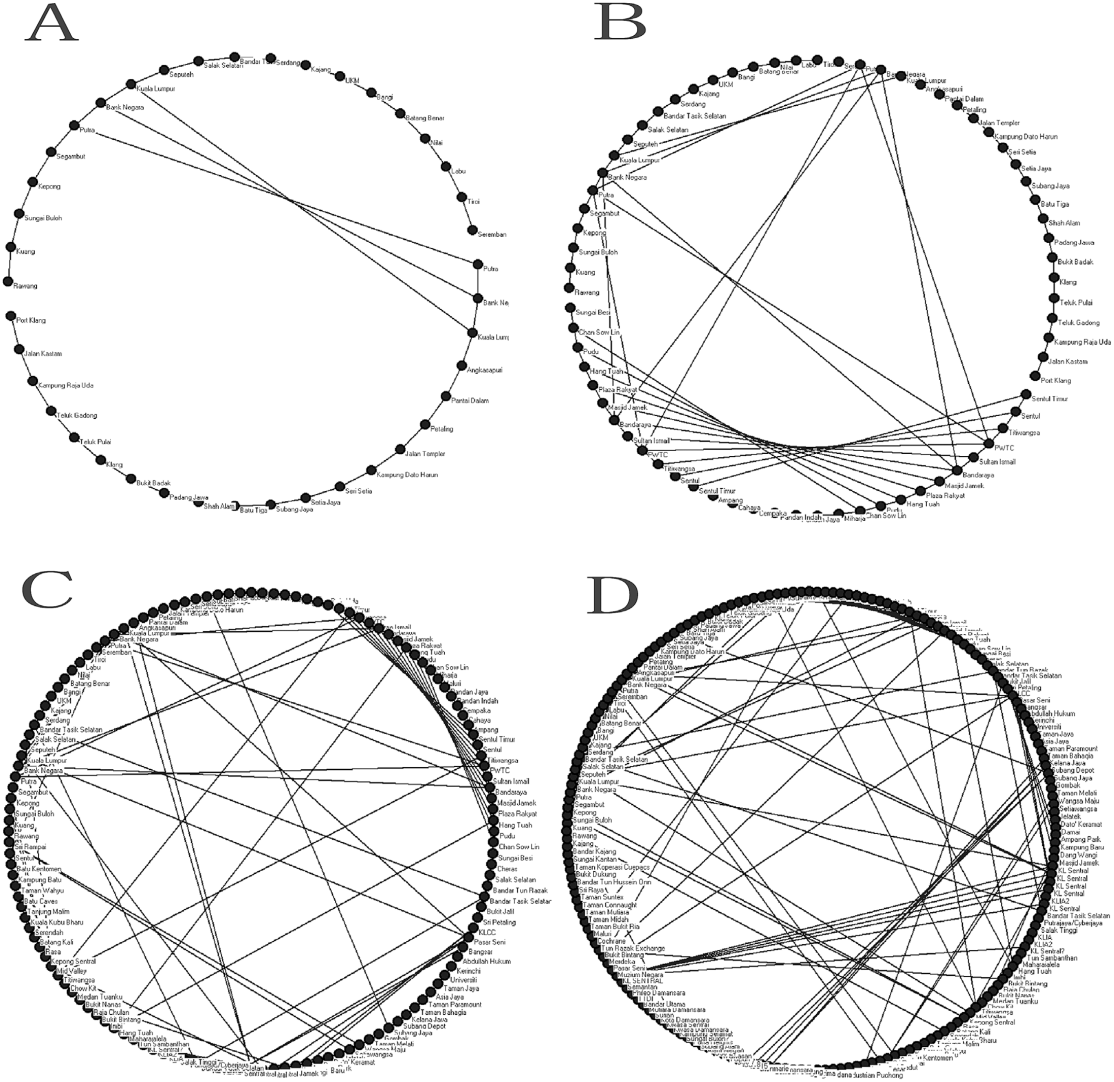
The topological structure of the PURTNoKL in different years. (A) G1995. (B) G2003. (C) G2015. (D) G2017.

Fig 1 shows the basic network structure and network development plan of the PURTNoKL. Essential data from this network growth process were analyzed using the space-L method (primary method) to illustrate topological performance. Fig 2 shows the evolution of the topological network structure via increases in the service area boundary and the addition of nodes. The weight and length of each edge was denoted as 1, which improved measurements and illustrated the importance of the nodes topologically. The same station was provided a different notation for each different line; therefore KL Sentral station is seen on many lines. Unlike certain previous theories, this assumption explain the performance of a single node in various lines.

Next, we simulated the network growth process by adding different numbers of nodes to observe the dynamic changes in the network and to identify areas that are relatively stable and unaffected by change. These stations are likely to be considered potential growth points. Finally, we used artificial methods to attack the network to determine its robustness.

### 3. 1 Network representation

Using the popular graph theory, the PURTNoKL can be represented as an undirected connected network *G* = <V, E>, where V is the set of nodes, N is the number of nodes when V = {v_i_ |i ∈ I ≡ {1, 2,…, N}}, E is the unordered pairs or edges of elements of V and is denoted by e_ij_, and E = {e_ij_ = (v_i,_ v_j_)|i, j ∈ I}. In addition, the number of edges is denoted as M. The adjacency matrix of networks is A = [a_*ij*_]_*n*×*n*_, representing the connection between nodes v_i_ and v_j_, which is defined as ${\text{a}}_{\text{ij}}=\left\{ \begin{array}{l} 1,(vi,vj)\in E\\ 0,(vi,vj)\notin E \end{array}\right.$, where a_ii_ = 0 to remove any self-connections. In addition, A = [a_*ij*_]_*n*×*n*_ is symmetrical and non-negative. G_2017_ denotes the rail transit network system in 2017.

### 3. 2 Network indicators

#### 3.2.1 Classical traffic indicators

Here, we choose the number of nodes and edges, the complexity, the connectivity, the network loops and the availability of loops as related indices. The ratio between M and N, $\beta =\frac{M}{N}$, reveals the complexity of network growth. Similarly, connectivity—a connection indicator proposed by Kansky [3]–measures the ratio between the number of actual edges and M. The total number of edges possible in a plane, M_max_, can be calculated through the deduction of Euler's formula and is represented as *M*_max_ ≤ 3*N*−6. Therefore, the connectivity index can be defined as $\tau =\frac{M}{3N-6}$. As per graph theory, the loops in a network can be calculated by the formula presented by Ore [31]: *M*_loops = *M*−*N* + 1_. The availability of loops is also introduced by Kansky [3]. This value is calculated as the ratio between the number of existing loops and the total possible number of loops in a plane, $a_{loops}=\frac{M-N+1}{2N-5}$.

#### 3.2.2 Complex network indicators

There are many indicators in complex network theory. Here, we present indicators that illustrate the topological network characteristics and their spatial implications for transit networks using three categories: connection, clustering, and centrality.

Connection indicators. The degree of connection k_i_ is defined as the connection of nodes, suppose that node 1 only have one link with node 2, then *k*_1_ = 1. The average degree of connection is $<k>=\frac{2M}{N}$; the total degree of network connection is denoted as TD. Degree distribution stands for the probability of randomly choosing node v_i_ when k_i_ equals k and is denoted as P(k). The cumulative degree distribution is defined as $P_{k}=\sum_{k\prime =k}^{\infty }P(k\prime )$; in other words, it is the fraction of nodes with degree values that are not less than degree value k. Given two nodes, *v*_*i*_, *v*_*j*_ ∈ *V*, let $d_{\text{min}}^{ij}$ be the shortest path length between them; we can then define the longest path length between these two nodes as the network diameter, D. The average path length of the network is then described as $APL=\frac{2}{N(N-1)}\sum_{i\neq j}^{}d_{\text{min}}^{ij}$. In terms of the efficiency of the global network [14], E(G) is the inverse of the shortest path length between each pair of nodes v_i_ and v_j_ and is computed as $E(G)=\frac{2}{N(N-1)}\sum_{i\neq j}\frac{1}{d_{\text{min}}^{ij}}$; it shows the average efficiency of transit flow or information between nodes in the network.

Clustering indicators. Watts and Strogatz [32] introduced the clustering coefficient to characterize the degree of clustering in a network. This coefficient is a measure of the extent to which a node, v_i_, shares neighbors with other nodes, which is defined as $C(v_{i})=\frac{2e_{i}}{m_{i}(m_{i}-1)}$, where e_i_ is the number of edges shared with local neighbors of node v_i_, and m_i_ is the connection degree of local neighbors of node v_i_. C(v_i_) is derived from the unit interval 0 to 1, which is called the local clustering coefficient. The global clustering coefficient is denoted as $C(G)=\sum_{i=1}^{N}C(v_{i})/N$. The K-core partition is a concept introduced by Seidman [33] from the field of social networks to represent the evolution of a network and to depict an area that has a crucial influence on that network. In some extraordinary examples, it can also explain why some nodes have a greater degree of connection but are less important in a network. The k-core of graph G is a maximally connected subgraph in which all vertices have at least degree k.

Centrality indicators. The degree of centrality is defined as $DC(v_{i})=\frac{ki}{N-1}$, or the number of links incident on a node that can reflect the importance of the node v_i_ in relation to spatial geography, which indicates that a node with more neighbors is more important in a network. Closeness centrality (C_closeness_) is denoted as the reciprocal of the average distance between each node *d*_*ij*_; where $d_{ij}=\frac{\sum_{i\neq j}d_{ij}}{N-1}$ is defined and can be obtained using $C_{closeness}(v_{i})=\frac{1}{d_{ij}}=\frac{N-1}{\sum_{i\neq j}d_{ij}}$. This index means that if a node is closer to other nodes, it is more important in the network; it describes the relative location of a node. Betweenness centrality (C_betweenness_) was originally defined by Freeman [34] as the total number of shortest paths between two separate nodes d_min,st_ and passing through node v_i_; it reflects the load on node v_i_ and can alternately understood as the controllability of the node. The more routes passing through a node, the easier it is to control the flow of transit to other nodes. On this basis, centrality can be clarified as $C_{betweenness}(v_{i})=\sum_{i\neq s\neq t\in V}\frac{d_{\text{min},st}^{i}}{d_{\text{min},st}^{}}$, and the normalization of betweenness is described as $BC(v_{i})=\frac{\sum \frac{d_{\text{min},st}^{i}}{d_{\text{min},st}}}{\frac{\left(N-1)(N-2\right)}{2}}=\frac{2\sum \frac{d_{\text{min},st}^{i}}{d_{\text{min},st}}}{\left(N-1)(N-2\right)}$, where $\frac{\left(N-1)(N-2\right)}{2}$ is the maximum possible value.

### 3.3 Cascading failures

When the rail network is affected by internal and external factors, the capacity of some of the nodes or edges is exceeded. When this occurs, failure or error emerge. Because of combinations and connections with surrounding nodes and edges, failure and error are amplified and spread according to certain rules. This ripple effect eventually leads to the jam of part of a network or even the collapse of the entire network. This phenomenon is called cascading failure [35]. Most researchers focus on the connectivity and reliability of networks and test their robustness [16–21]. In this study, we also test the robustness of the PURTNoKL. Several related strategies are applied, including the node and edge elimination strategy, based on the network indices shown in section 3.2.

## Numerical Analysis

### 4.1 The description of the topological evolution process

#### 4.1.1 Steady increase in network size

**Fig 3:**
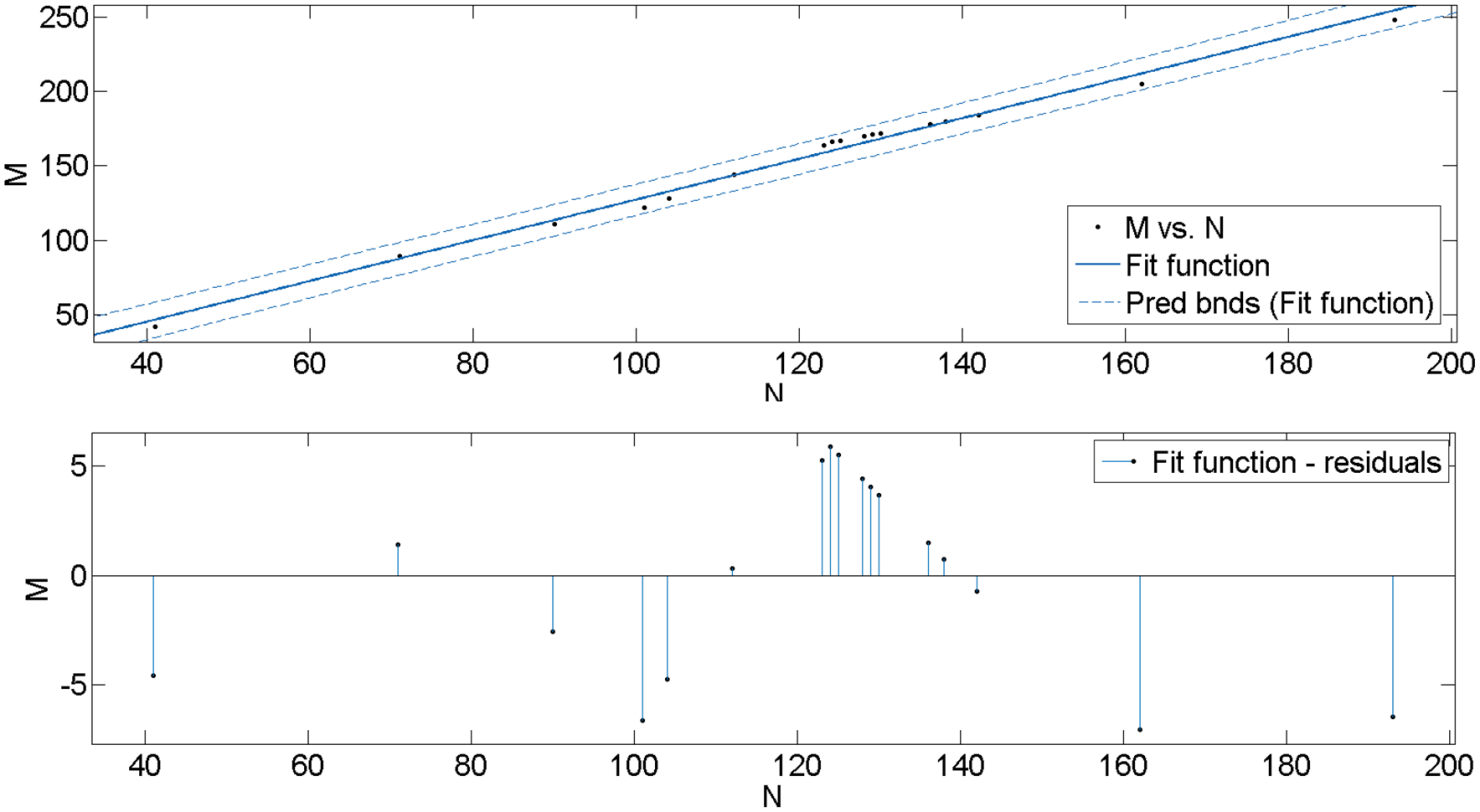
The relationship between N and M.

Based on the related functions described in sections 2 and 3, the basic properties and topological characteristics of PURTNoKL are provided in Table 1, excluding the centrality of nodes, which will be analyzed in the next section. Fig 3 shows the relationship between the number of nodes N and the number of edges M in the network. The results presented in Table 1 clearly define a linear relationship between these two indices; the fitting function for the line is *y* = 1.4*x*−9.5, *R* ^2^ = 0.9905 (Fig 3), and there is a high fitting confidence coefficient. This function illustrates that the growth of nodes and the connections between them obey a linear rule, which lays the foundation for a broad network prediction.

**Table 1:** The basic topological characteristics of PURTNoKL.

Year	N	M	β	τ	M_loops_	a_loops_	<k>	TD	APL	D	E(G)	C(G)
1995	41	42	1.0244	0.3590	2	0.0260	2.0488	84	10.9220	31	0.0916	0.0732
1996	71	89	1.2535	0.4300	19	0.1387	2.5070	178	10.7911	32	0.0927	0.1070
1998	90	111	1.2333	0.4205	22	0.1257	2.4667	222	10.3700	32	0.0964	0.1122
1999	101	122	1.2079	0.4108	22	0.1117	2.4158	244	11.0188	32	0.0908	0.1000
2001	104	128	1.2308	0.4183	25	0.1232	2.4615	256	11.2018	33	0.0893	0.1019
2002	112	144	1.2857	0.4364	33	0.1507	2.5714	288	10.4318	32	0.0959	0.1161
2003	123	164	1.3333	0.4518	42	0.1743	2.6667	328	10.0376	32	0.0996	0.1235
2004	124	166	1.3387	0.4536	43	0.1770	2.6774	332	9.9920	32	0.1001	0.1326
2006	125	167	1.3360	0.4526	43	0.1755	2.6720	334	10.0449	32	0.0996	0.1316
2007	128	170	1.3281	0.4497	43	0.1713	2.6563	340	10.2792	32	0.0973	0.1285
2008	129	171	1.3256	0.4488	43	0.1700	2.6512	342	10.3808	32	0.0963	0.1275
2009	130	172	1.3231	0.4479	43	0.1686	2.6462	344	10.4935	33	0.0953	0.1265
2010	136	178	1.3088	0.4428	43	0.1610	2.6176	356	10.6000	33	0.0943	0.1268
2011	138	180	1.3043	0.4412	43	0.1587	2.6087	360	10.8007	33	0.0926	0.1250
2015	142	184	1.2958	0.4381	43	0.1541	2.5915	368	10.9065	33	0.0917	0.1214
2016	162	205	1.2654	0.4271	44	0.1379	2.5309	410	12.2693	33	0.0815	0.1003
2017	193	248	1.2850	0.4328	56	0.1470	2.5699	496	12.2127	33	0.0819	0.0908

**Fig 4:**
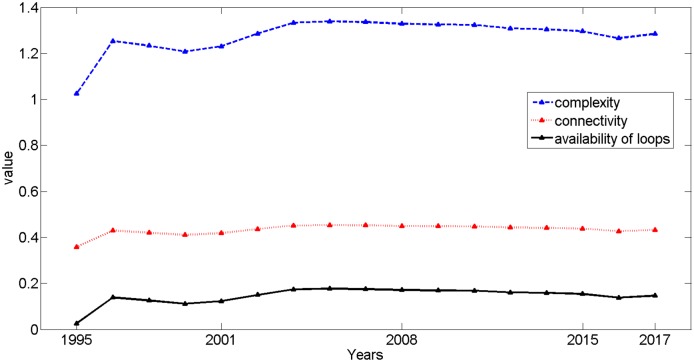
The changing trends of network complexity, connectivity, and availability of loops.

**Fig 5:**
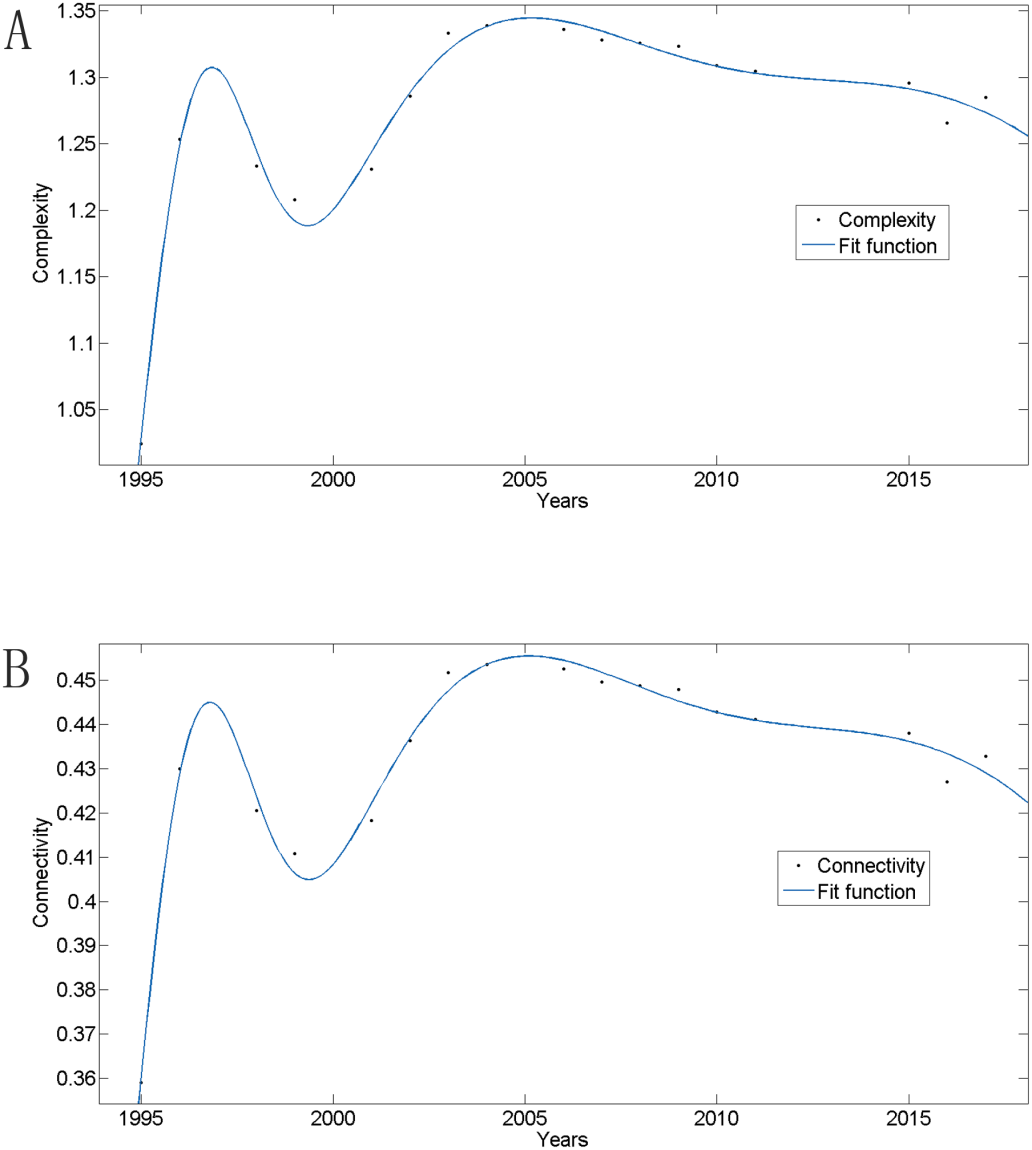
Fit line. (A) Network complexity, (B) Connectivity.

Fig 4 shows that network complexity β, connectivity τ and availability of loops a_loops_ follow a similar trend, and all have fluctuated over the past 17 years. More specifically, there was an initial dramatic increase from 1995 to 1996, and record high values were attained in 2004. Afterwards, all values have declined gradually. For network complexity β, G_1995_ equalled 1.0244, illustrating that the network forms a nearly tree-like structure. Generally, β fell within the interval [1.0244, 1.3387]. When this occurs, we find that β complies with the general Gauss model, and a function could be better fit as:

f(x) = 0.5678*exp(-((x-4.784)/7.111)^2) + 1.245*exp(-((x-17.15)/15.41)^2)+ 0.29*exp(-((x-1.961)/1.418)^2),

when R-square = 0.9894. Connectivity τ had a line that fit similarly, as shown below (Fig 5). Network diameter D increased from 31 to 33 and remained 32 from 1996 to 2008. The loops M_loops_ grew from 2 to 43 from 1995 to 2004 and then remained steady at 43 until 2015. This value is expected to reach 56 by 2017. These indices have shaped the PURTNoKL at the graph level and illustrate a fundamental growth trend across the network. When the network variations from before and after the node additions are analyzed, we can calculate the impact of these nodes and provide useful guidance for future rail line site selections.

#### 4.1.2 Degree distribution obeys a heavy-tailed distribution

**Fig 6:**
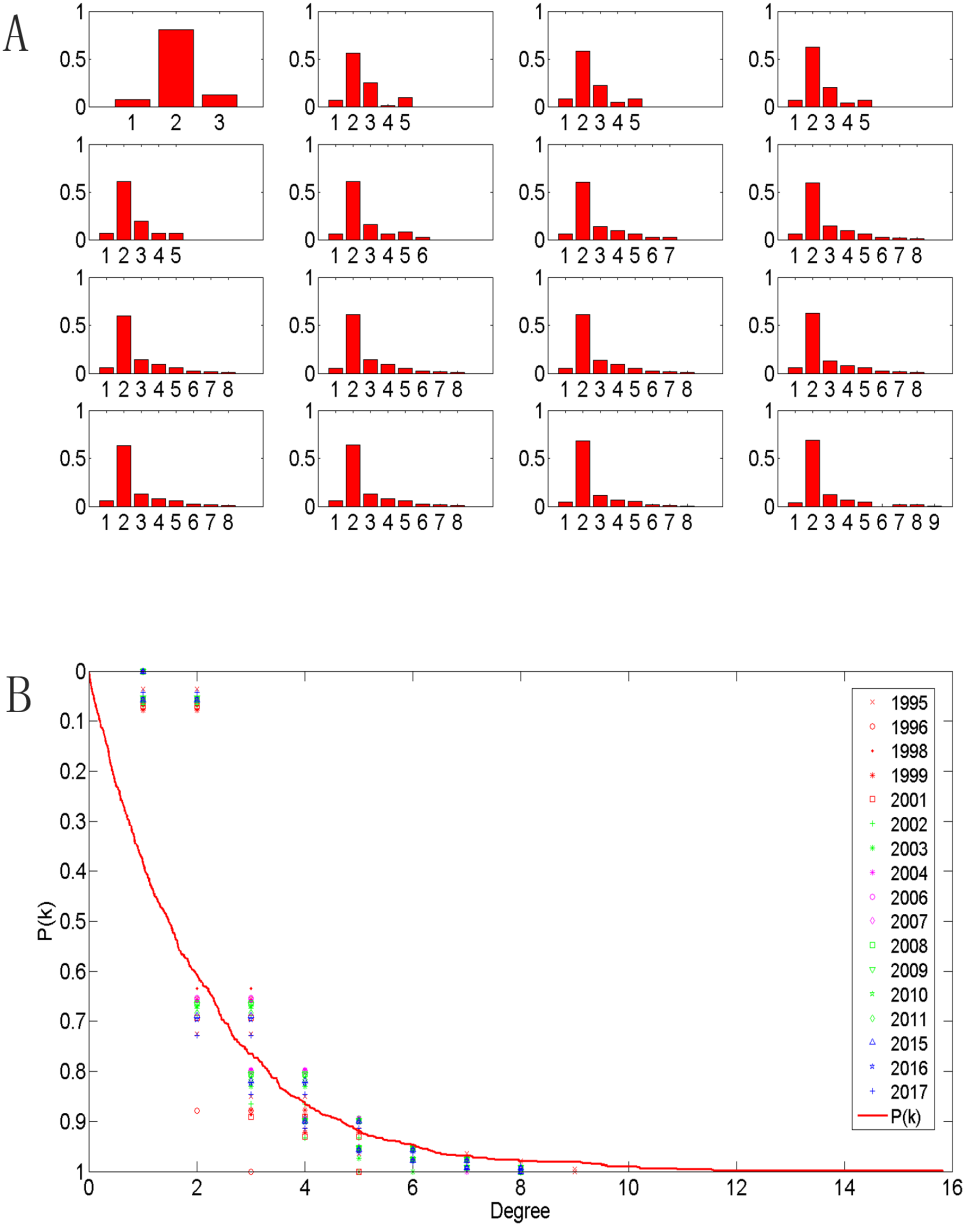
Degree distributions follow a heavy-tailed distribution. (A) Degree distribution: the horizontal axis represents degree value, and the vertical axis represents degree distribution; (B) CPD of degree.

Fig 6A shows that the degree distribution of PURTNoKL is conspicuous. In other words, these nodes with a degree value of 2 occupy a high proportion within existing nodes; at each phase of development their proportion was greater than 57%, and this value will reach 69% (at its peak) by 2017. At the network-generating phase (G_1995_), the largest node degree was only 3, but this figure will increase to 9 by 2017. This increase illustrates that the link between two nodes is primarily related from the same rail lines; and the level of intensive network development cannot keep pace with the speed of network expansion in the plane. Since 2002, the distribution pattern has stabilized, as in a heavy-tailed distribution. The degree’s cumulative probability distribution (CPD) was calculated and is shown in Fig 6B. It fit an exponential distribution and is defined by the function $P(k)=\mu_{1}e^{-\mu_{1}x}$. The scaling factor μ_1_ falls within [1.8, 2.6] and here is *μ*_1_ = 2. The average degree <k> was also calculated. According to the data, the average degree falls within the interval [2.0488, 2.6774], and the arithmetic mean is 2.5506. This index increased from 1995 to 2004, when it peaked. Since that time, it fluctuated slightly but slowly decreased; and it will reach 2.5309 in 2016 and 2.5699 in 2017.

**Fig 7:**
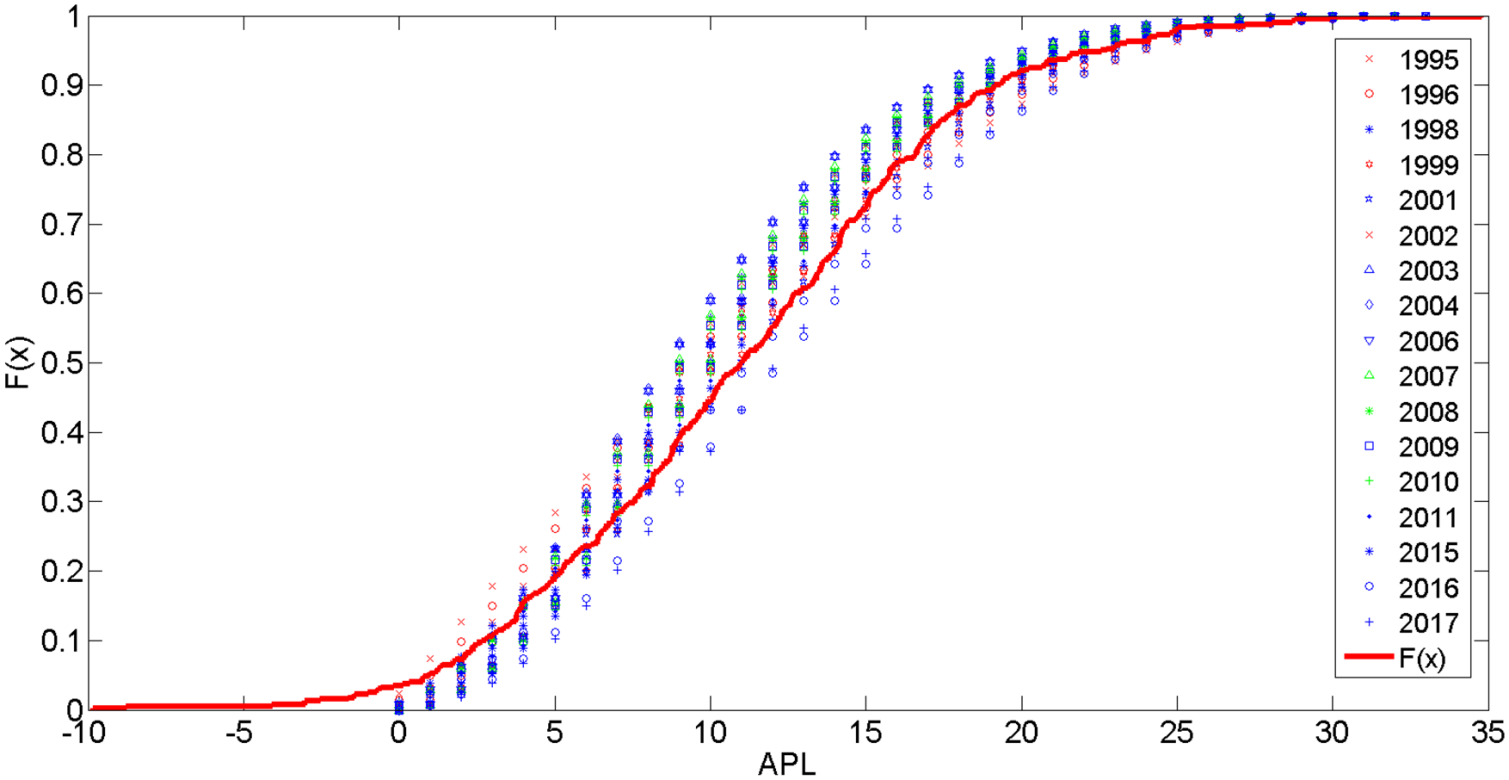
The CPD of APL.

As Table 1 shows, the minimum APL of G_2004_ was 9.9920. The APL will increase to 12.2693 in 2016, when it will reach its highest value. Fig 7 shows that the CPD of the APL is best fit by a normal distribution function, depicted as $F(X;\mu_{2},\sigma_{2}^{})=\frac{1}{\sigma_{2}\sqrt{2\pi }}\text{exp}\left(-\frac{{\left(x-\mu_{2}\right)}^{2}}{2\sigma_{2}{}^{2}}\right)$, and the scaling factors *μ*_2_ ϵ[10.6556,12.1494] and σ_2_∈[6.1808,7.0045]. *μ*_2_ = 11 and σ_2_ = 6.6 are illustrated in Fig 7. By contrast, the efficiency of the network E(G) varies inversely with APL and peaked at 0.1001 in 2004. Consequently, with a higher value of APL, and the network become less efficient.

#### 4.1.3 Networks have definite cluster and centrality tendencies

**Fig 8:**
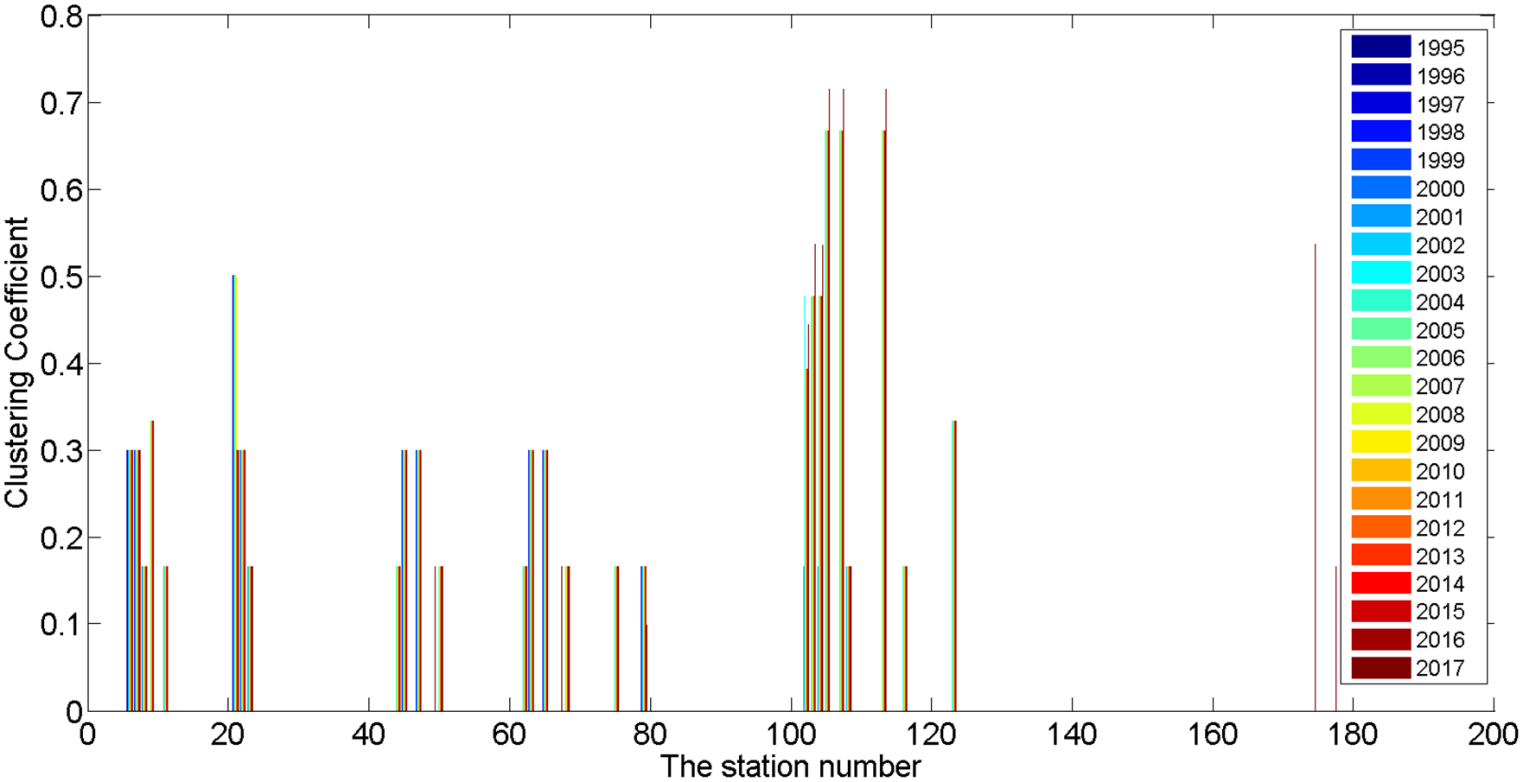
Clustering Coefficient partition of PURTNoKL.

**Fig 9:**
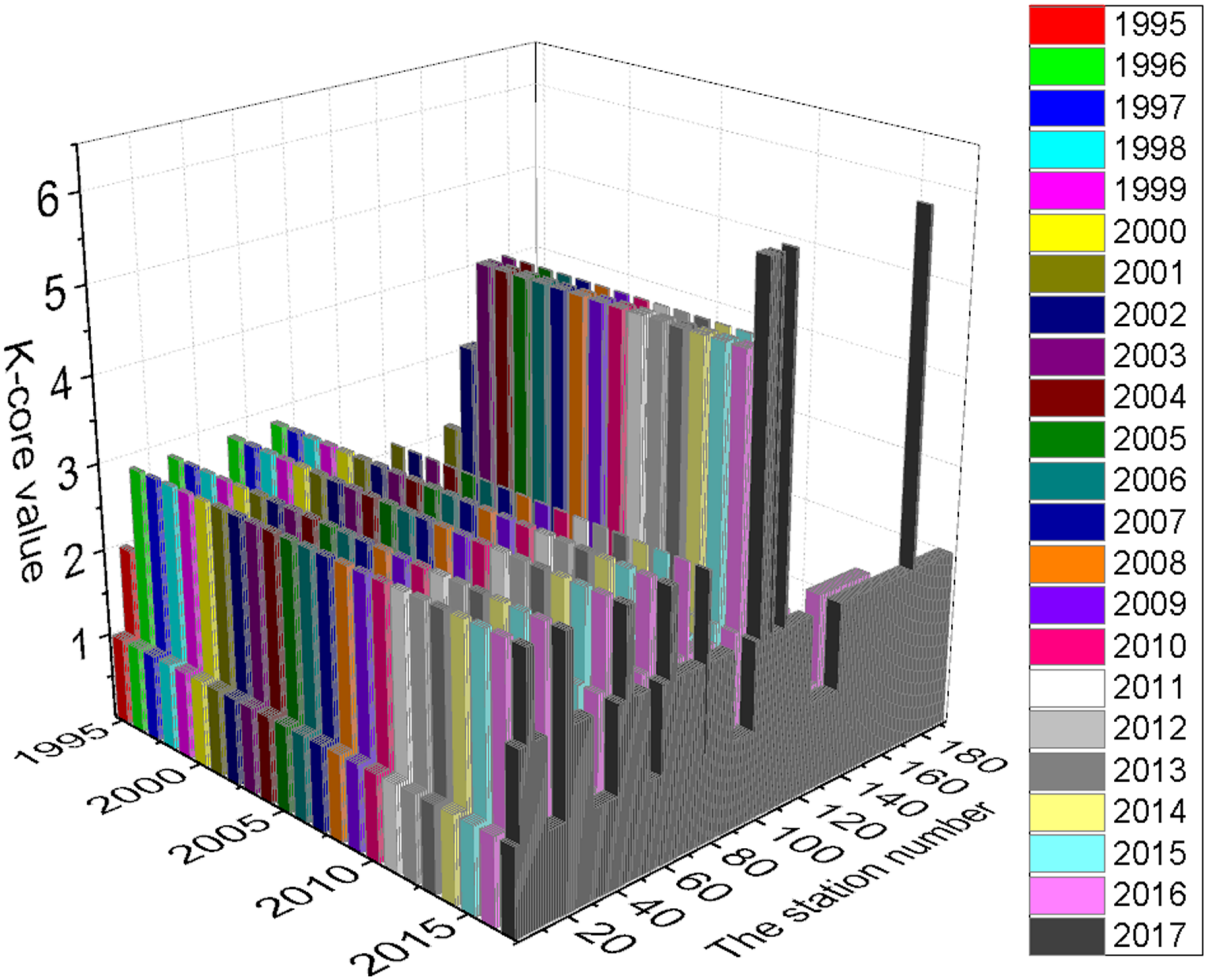
K-core partition of PURTNoKL.

This concept is a measure of the extent to which a node v_i_ shares neighbors with other nodes. Table 1 indicates that with the expansion of the network, the Global Clustering Coefficient C(G) increased from 0.0732 to 0.1326 and peaked in 2004. It has since decreased and will reach 0.0908 in 2017. In other words, the PURTNoKL had its tightest topological structure in 2004, and the structure has become looser since that time. Fig 8 shows the variation in the Clustering Coefficient C(v_i_). In general, the entire network clusters around 5 points; the most distinct cluster is centered on KL Sentral station, which is indicated by station numbers 100 to 120. The k-core partition reveals a similar clustering expression; most of the nodes fall within the 2-core, and KL Sentral station has the largest k-core value (see Fig 9).

**Fig 10:**
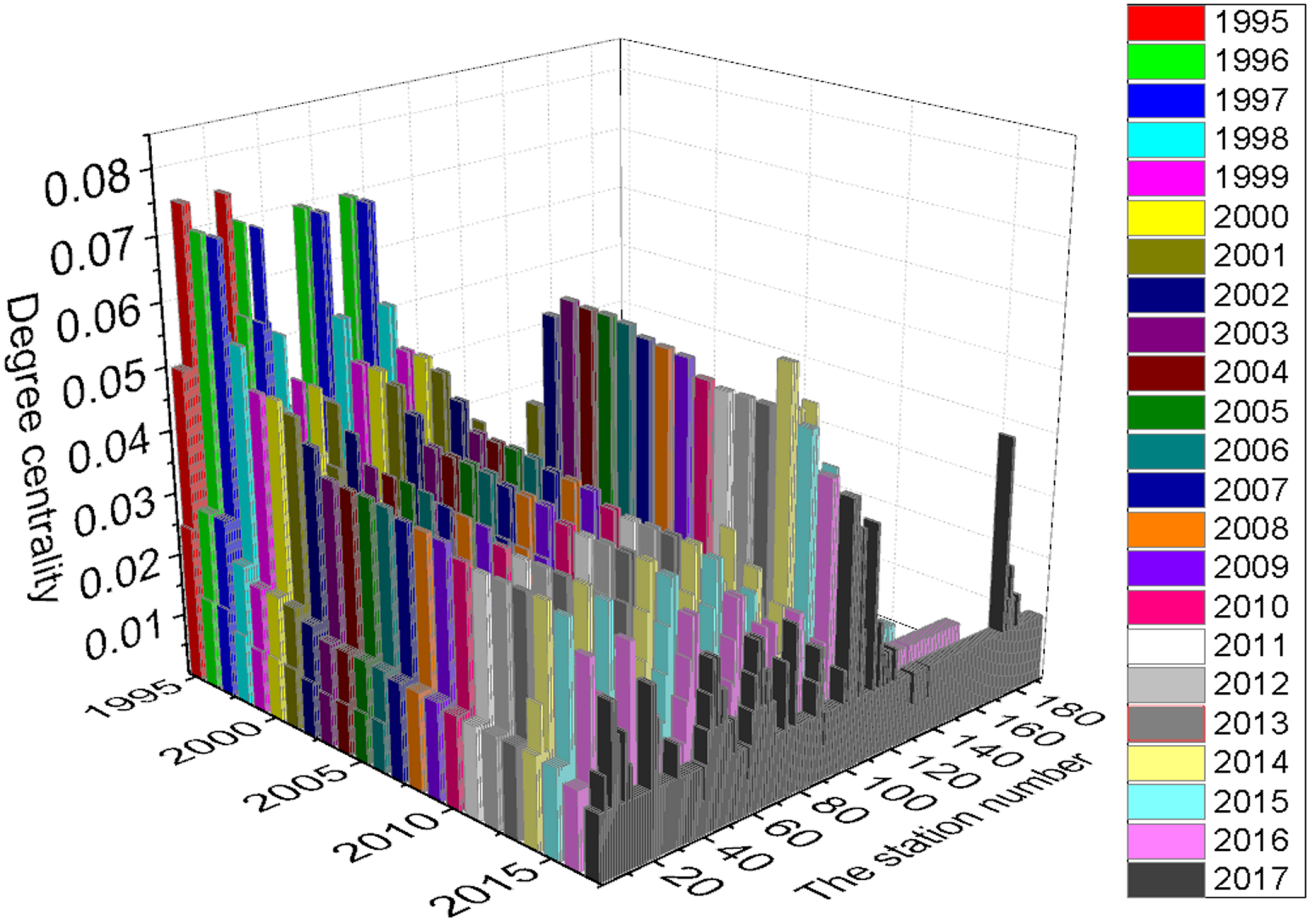
Degree of centrality partition of the PURTNoKL.

The total average degree of centrality is 0.0213; and the average G_2017_ is 0.0134. Unlike the k-core, most nodes with values of 1-core and 2-core have the same degree of centrality (Fig 10). As the network grows, nodes that once had a considerable degree of centrality become less important. In 2017, two important areas will develop higher k-core values and experience a change in their degree of centrality: Bank Negara—Kuala Lumpur—KL Sentral—Seputeh—Salak Selatan (station numbers 7 to 11), and Bandaraya—Masjid Jamek—Plaza Rakyat (station numbers 47 to 49).

**Fig 11:**
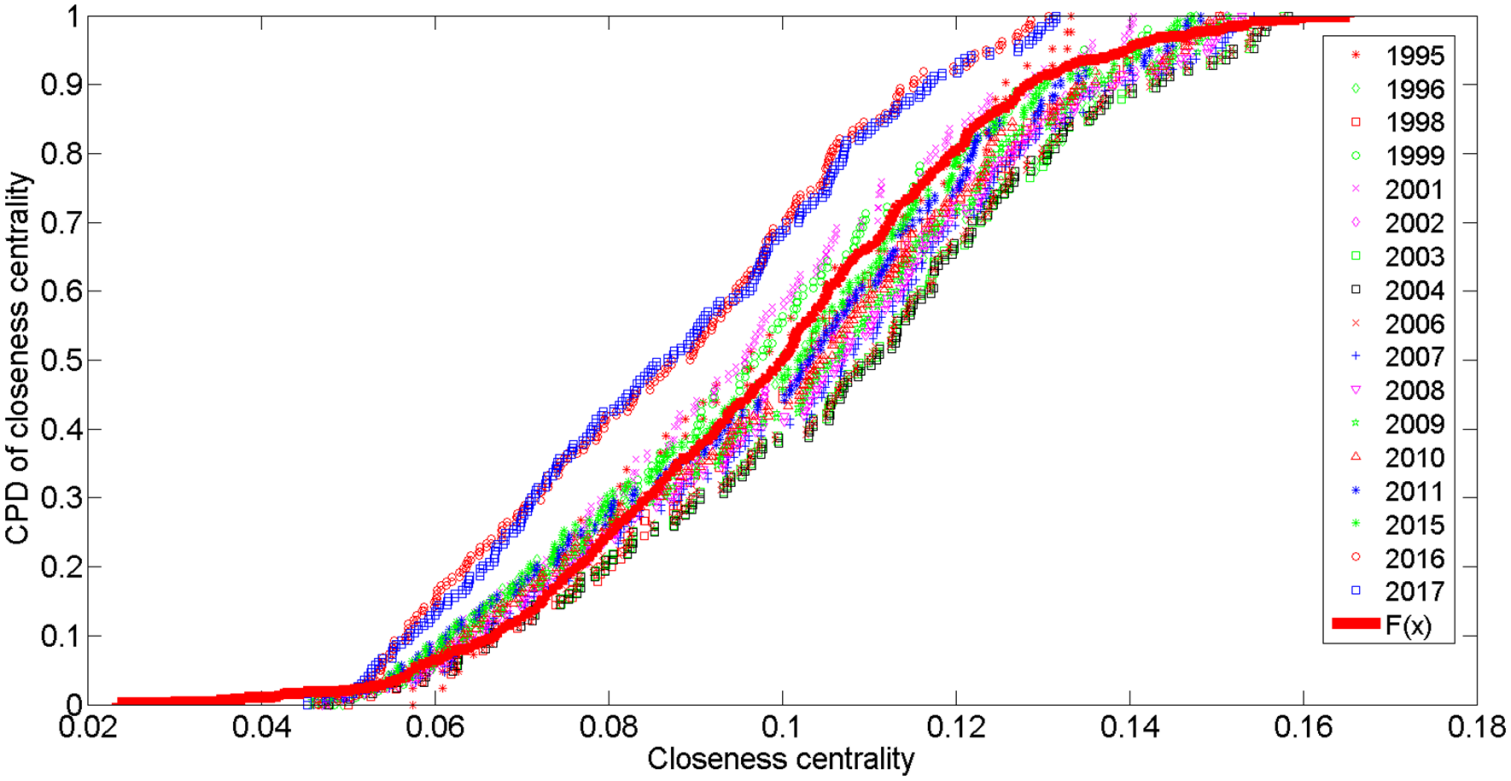
The CPD of closeness centrality for the PURTNoKL.

The average closeness value for the network was 0.0991 and will reach 0.0870 in 2017. The cumulative probability distributions of closeness centrality are best fit by a normal distribution function, depicted as $F(X;\mu_{3},\sigma_{3})=\frac{1}{\sigma_{3}\sqrt{2\pi }}\text{exp}\left(-\frac{{\left(x-\mu_{3}\right)}^{2}}{2\sigma_{3}{}^{2}}\right)$, where the scaling factors are *μ*_3_ ∈[0.0870,0.1067] and d *σ*_3_∈[0.02221,0.02738]. We chose *μ*_3_ = 0.098 and *σ*_3_ = 0.025 to illustrate this function in Fig 11. The larger the index value, the greater the impact or service area. This value can also be employed to estimate the prevalence of traffic congestion in a network [36, 37]. In general, the lowest closeness centrality values will be recorded in 2017; however, as discussed above, the network clusters around five points. We should note that with the operation of a new line in 2017, the area linking Surian—Mutiara Damansara—Bandar Utama—TTDI—Phileo Damansara will undergo improvements in regional development because this area will have increased in closeness centrality (the average value will be approximately 0.1).

**Fig 12:**
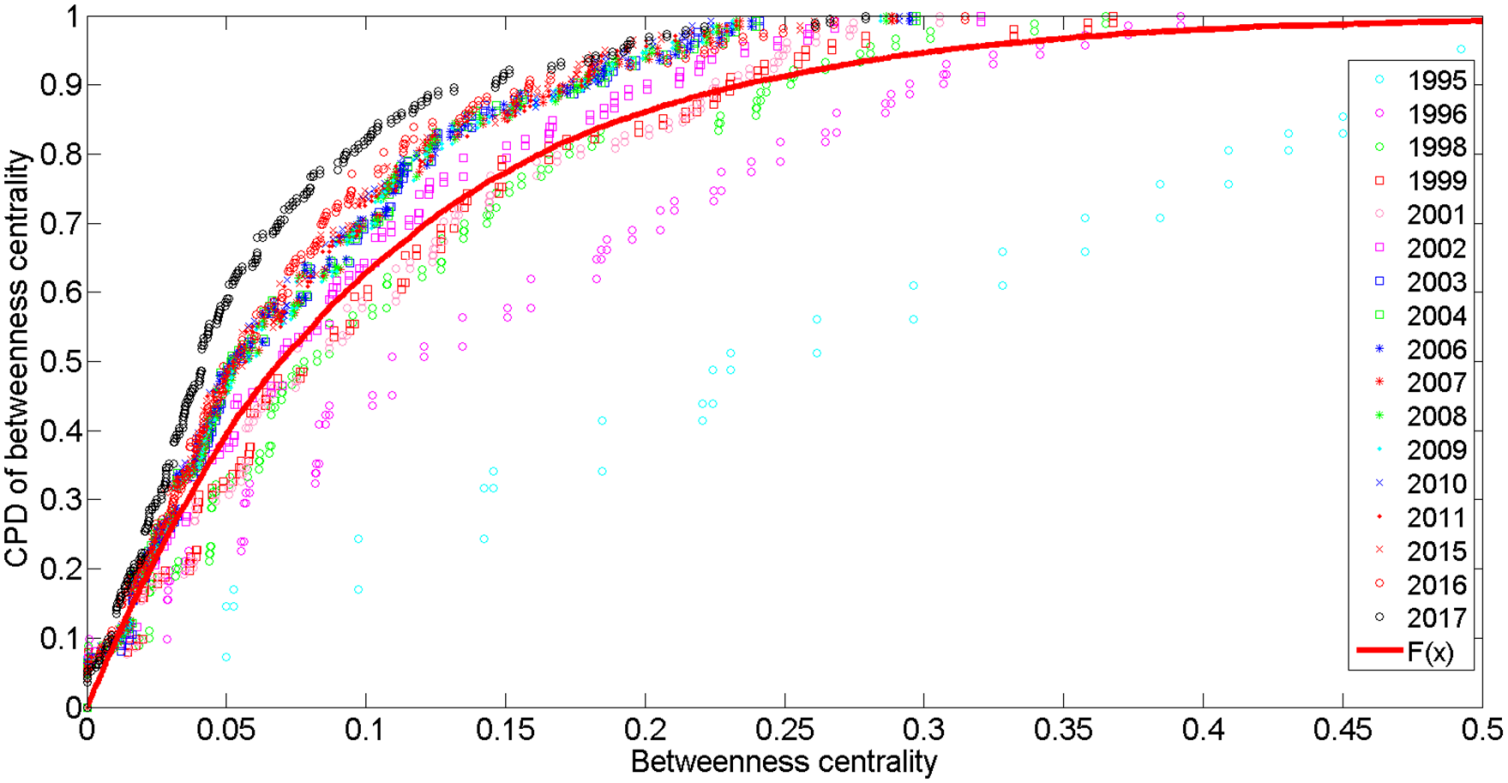
The CPD of betweenness centrality for the PURTNoKL.

Using the same data measuring method, we found that the average betweenness centrality value was 0.0836, and the maximum value, 0.5216, occurred in 1995 at Kuala Lumpur station. At that time, the network consisted of only two lines. Therefore, a central node was able to better control all transit in the network. The data show that the value of KL Sentral Station is 0.2790 in 2017, which corresponds to the real situation. The CPD of betweenness centrality was calculated and is shown in Fig 12. An exponential distribution best fit these data, which is described by the function $P(k)=\mu_{4}e^{-\mu_{4}x}$ and scaling factors *μ*_4_ ∈[0.0593,0.2544]. We selected μ_4_ = 0.1 for modelling purposes. In 1995, CPD formed a nearly straight line because the network mirrored a tree structure and was less complex. The changing trend shows that with the expansion of the network, μ_4_ becomes smaller; however, an increase in nodes reduces the betweenness centrality.

### 4.2. Network growth

Prior to this work, planners mainly considered the traditional effects of adding nodes to a network; these effects included the influence of nearby nodes on traffic diversion, the cost of time and surrounding land prices. Our research findings suggest that another effect be considered. Based on the topological network structure and related indices, four typical growth styles affect the PURTNoKL, and the optimization growth style requires a different strategy.

#### 4.2.1 Four growth styles

**Fig 13:**
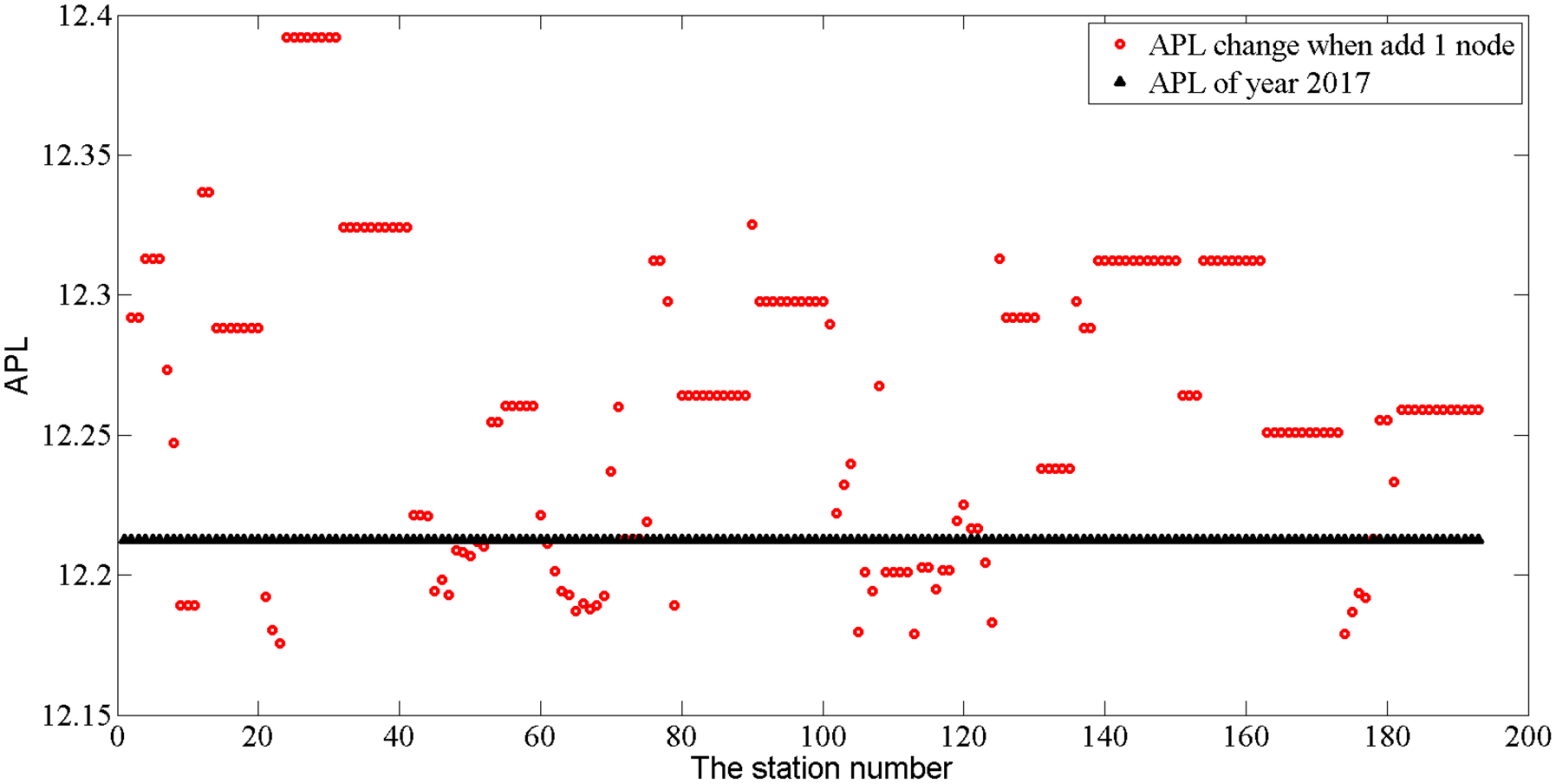
The changing trend in APL when a single node is added to the network.

The first kind of network growth style is single station add-on. By adding nodes to the network, it has been found that nodes can work separately as push-nodes and pull-nodes. The addition of a push-node may decrease APL (here, we selected APL as the measurement indicator because APL is the inverse of E(G), the efficiency of the global network), which would make the network more efficient. This efficiency was seen when KLCC and Pasar Seni stations were added in 1998, and when the Mid Valley station was added in 2004. By contrast, pull-nodes (e.g., Kepong Central station, added in 2006) cause the network to become less efficient (see the calculations shown in Table 1). Table 1 and Fig 13 illustrate that when we add one node to G_2017_, APL changes dramatically; adding nodes to particular stations or lines will affect the network structure and make the network run much more inefficiently (e.g., APL increases, lies above the black triangle line) or more efficiently (below the line). When APL decreases, there is the potential for network extension.

The next style is new line added. The construction and operation of the KLIA Express, KLIA Transit and KL Monorail Lines increased the complexity, connectivity and other indices after 2002 and 2003. This history illustrates that adding new lines may benefit the network and is supported by predictive data for G_2017_, when an entire new line will be added.

Connect related lines. In 2001, the KL Sentral station was constructed to connect the Seremban, Port Klang, and Kelana Jaya Lines. In 2002, the Bandar Tasik Selatan station was built to connect the Seremban Line with the Sri Petaling Line. This process yields a more complex network structure, and the connecting stations become vital transfer stations that allow people to efficiently change lines.

Line extensions, such as those that occurred on the Sri Petaling and Kelana Jaya Lines resulted smaller connectivity, and larger APL values, additionally, the topological structure became simpler and less efficient. Most importantly, by adding select individual nodes at the end of the rail line, the network change becomes a line extension linked by a single station add-on.

**Fig 14:**
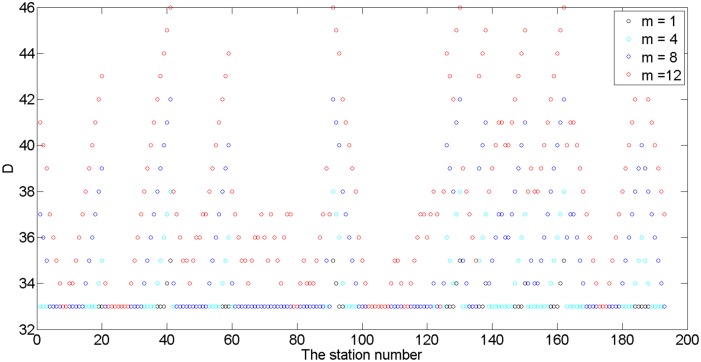
The changing trend in D when 1, 4, 8, 12 nodes are added in.

We investigate what would occur when we added different numbers of nodes to the system based on G_2017._ We used m to count the nodes that add in. When m = 1, m = 4, m = 8 and m = 12, we observed dynamic changes in D and identified specific areas that were relatively stable and unlikely to be significantly affected by the addition of the nodes. These points have the greatest potential for network extension—even after adding a new line—because adding nodes to at these points will not change the diameter of a network. Fig 14 indicates that stations on the Port Klang Line (Putra to Setia Jaya, stations 21 to 30, station list can be see Table in S1 Table) and the Sungai Buloh-Kajang Line (the points near KL Sentral station, stations 170 to 180) have a more stable structure. The data also indicate that 8 nodes on the Ampang Line (Sentul Timur to Sungai Besi, station 60 to 70), the Sri Petaling route (Cheras to Sri Petaling, station 72 to 77) and the Kelana Jaya Line (KLCC to Subang Jaya, station 78 to 90) have extension potential.

Most of the points with higher extension potential are located near the city’s economic center, meaning that the rail networks may contribute to the rich-get-richer phenomenon. In general, they obey the degree of preferential attachment mechanism. We also could selected m = 12 based on the APL of the network, selected particular nodes as fixed nodes, and identified every extension line that passed through those nodes to determine the changing trend. This type of extension may be treated as a new line added in or as a connection to related lines.

#### 4.2.2 Network growth trend

Based on the situations discussed above, we maintained an unchanged numbers of initial nodes for G_2017_ and identified six different strategies to simulate the growth process. Strategy 1 requires the calculation of total network betweenness centrality for each node and connects the nodes with the smallest betweenness centrality values. If more than one pair of nodes has the lowest values or are already linked, another pair with the same small value is randomly chosen and a new network is created. Then, the new network is recalculated for purposes of obtaining a new matrix of the smallest betweenness centrality values for every node, and new links are added following the same strategy. This process is run repeatedly until one-half of the total number of links are added, as measured by *f*_a_ = *M*′/*M* ^0^ = 0.5, where M' is the total number of new links added, and M^0^ is the total number of edges for G_2017_. Strategy 2 computes the shortest path lengths between each node, and then connects the two nodes with the largest value. If more than two nodes have the longest shortest path lengths, two of these are randomly selected and linked. As in strategy 1, the new network is recalculated and links are added until f_a_ is obtained. Strategy 3, as proposed by Huang and Chow [38], is based on strategy 2 and computes the pair of nodes p and q, which have the biggest shortest path lengths, where k_p_ and k_q_ are the degrees of nodes p and q, respectively; Q is the product of k_p_ and k_q_, and nodes p and q are connected if they have the smallest value of Q. Meanwhile if there is more than one such pair of nodes or these two nodes are already linked, another pair is randomly chosen. The new network is then recalculated and links are added until f_a_ is obtained. Strategies 4, 5 and 6, are based on the smallest degree, the most insignificant clustering coefficient and the most diminutive closeness centrality of nodes, respectively; and each connects the nodes with the lowest value, and then follows the same procedure as outlined in Strategy 3.

**Fig 15:**
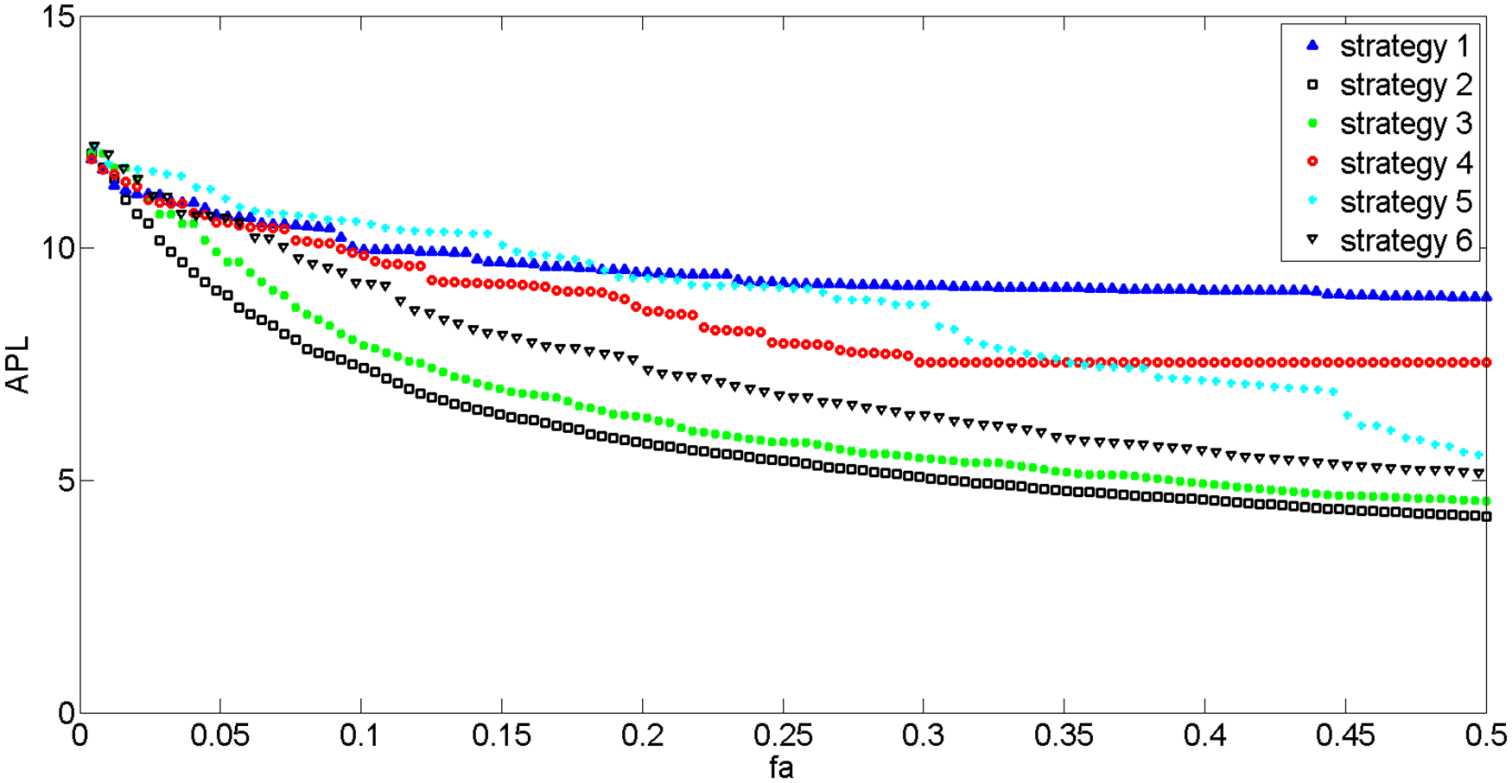
The changing trend of APL with different network growth strategies.

**Fig 16:**
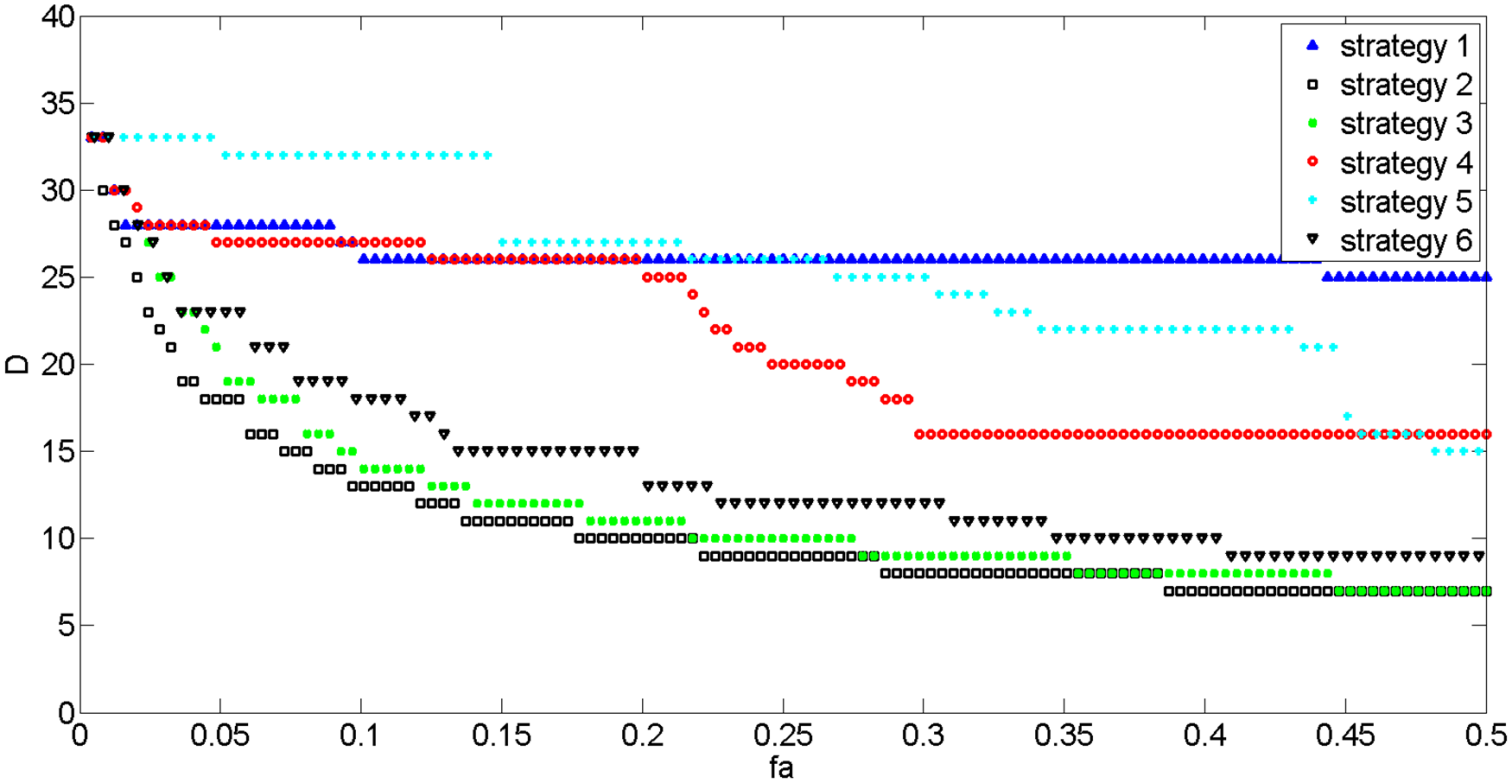
The changing trend of D with different network growth strategies.

Figs 15 and 16 show network growth following the 6 strategies outlined above. When links or edges are added to the network, APL decreases regardless of strategy; however, Strategy 2 indicates a significant decrease in both the APL and D of the network; when 10% of the total number edges is added, APL declines by 50% and D decreases by 60%. This result indicates that a network’s efficiency and character can be heavily affected by linking nodes with the biggest shortest path lengths; thus, it also suggests that the network should be expanded upon at these nodes.

### 4.3 Network failures

**Fig 17:**
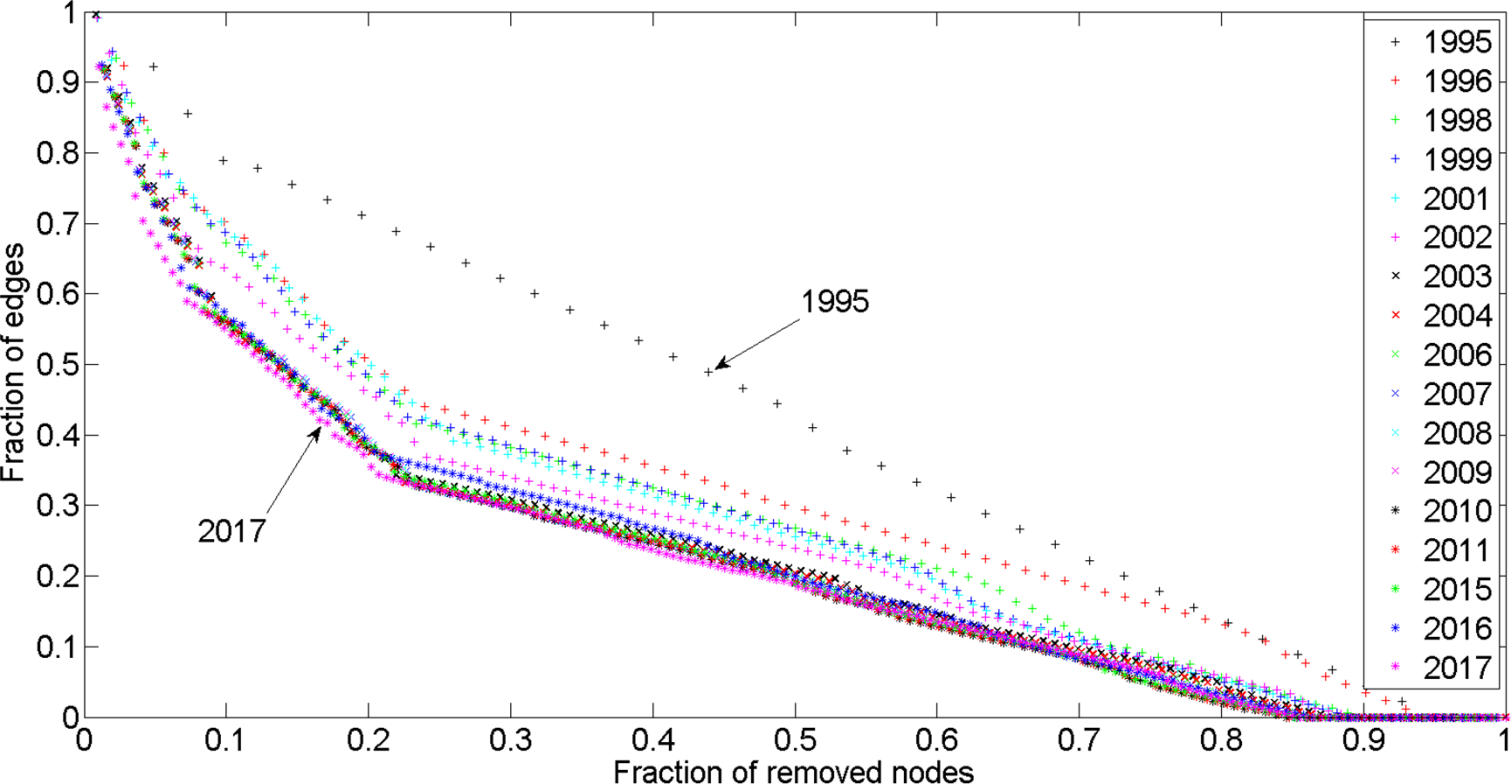
Network edges fraction change with largest degree-based attack.

Network cascading failures were investigated to measure the response of the rail network to different attack strategies. First we used the largest degree-based attack strategy. Analyzing a total of 17 networks from G_1995_ to G_2017_, we found that, during the expansion network process, the network became less robust, and 5% of existing nodes were removed (Fig 17), the total fraction of edges will drop approximately 40% by G_2017_. If this situation were to occur in real life, the entire rail network would rapidly be paralyzed. With the network’s growth, larger degree nodes occupy a smaller ratio of total network nodes. Removing these nodes would sharply decrease the network’s efficiency. When the ratio of the recalculated number of edges and the initial number of edges are set on the Y axes, we notice that, although this type of growth obeys the degree of preferential attachment mechanism and costs less, it does not benefit future development.

Next, we proposed several different attack strategies to analyze the changing trend of G_2017_. The 1st strategy focuses on the highest clustering coefficient and is based on the node elimination strategy. In other words, the node with the highest clustering coefficient is removed first, and the network is then recalculated to determine the new node with the largest value to be removed from operation. If two or more nodes have the largest value, one is randomly selected for elimination. The 2nd strategy is the largest node degree elimination strategy; it prioritizes the removal of the node with the greatest degree value. The 3rd strategy is the random attacks based on node elimination strategy. Here, one node is randomly selected and removed from G_2017_, and then the network’s performance is recalculated and the process is repeated. The 4th strategy is similar to the 3rd one; however, random attacks are based on the elimination of edges. The 5th strategy, which focuses on eliminating the node with the highest betweenness value, meaning that the node with the highest betweenness will be removed preferentially.

**Fig 18:**
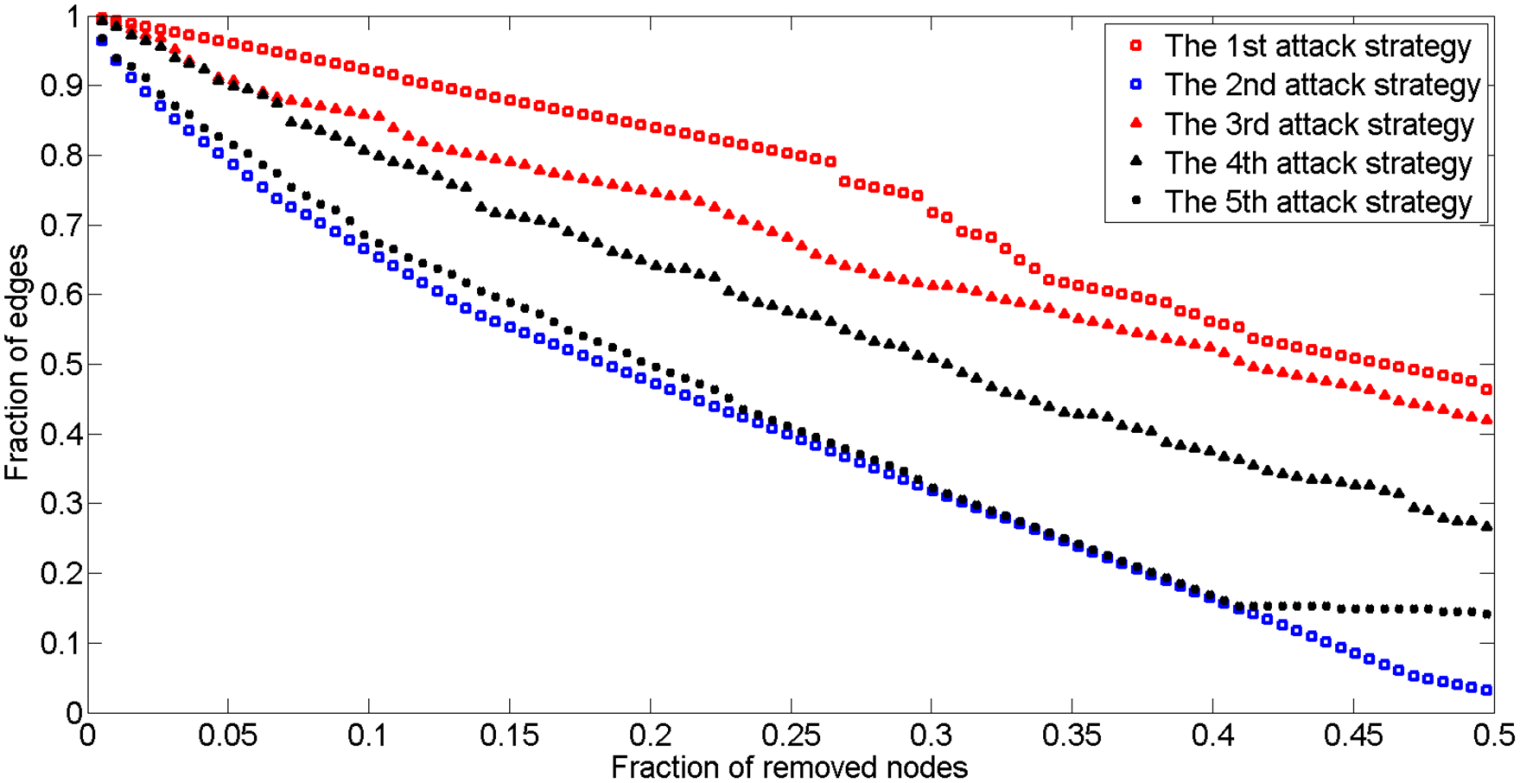
G_2017_ edge fraction changes with different attack strategies.

Fig 18 suggests that the largest node degree elimination strategy (Strategy 2) affects the G_2017_ most seriously, with 5% of the nodes removed and a 20% decline in the fraction of edges. However, the highest betweenness node elimination strategy can also effectively destroy the network. Therefore, to enhance network protection, additional investments and attention should be undertaken and paid to those nodes with the largest degrees and highest betweenness values.

## Conclusion

The construction of a topological network structure using complex network theory in this research reveals the network growth trend in the PURTNoKL. This novel approach to transit network mapping was based on an analysis of the network’s expansion with reference to the nodes with the biggest shortest path length values. It also prioritized network protection and revealed that those nodes with the largest degree and the highest betweenness values are most important to the network’s operation. These research findings have contributed to the design of the rail network. We also calculated the related network indices and topological network characteristics such as connection, clustering, and centrality. These calculations allow for a deep analysis and evaluation of rail and road networks, which may be useful in the urban planning processes of other countries, particularly as these relate to the analysis of traffic demand and the macro-analyses of local land-use). However, we did notice some shortcomings in the study. For example, we considered the network as an undirected graph and did not calculate the distance between each node or assess traffic flows.

## Supporting Information

S1 Table The list of stations. (PDF)

## References

[1] Chi G, Voss PR, Deller SC. Rethinking highway effects on population change. Public Works Management & Policy. 2006;11(1):18–32. 10.1177/1087724X06292336

[2] Gattuso D, Miriello E. Compared analysis of metro networks supported by graph theory. Networks and Spatial Economics. 2005;5(4):395–414. 10.1007/s11067-005-6210-5

[3] Kansky KJ. Structure of transportation networks: relationships between network geometry and regional characteristics: Ph.D. Thesis, University of Chicago; 1963.

[4] Garrison WL, Marble DF. Factor‐analytic study of the connkctivity of a transportation network*. Papers in Regional Science. 1964;12(1):231–8. 10.1111/j.1435-5597.1964.tb01269.x

[5] Garrison WL, Marble DF. The structure of transportation networks: Transportation Center at Northwestern University; 1961.

[6] Derrible S, Kennedy C. The complexity and robustness of metro networks. Physica A: Statistical Mechanics and its Applications. 2010;389(17):3678–91. 10.1016/j.physa.2010.04.008

[7] Quintero-Cano, Sayed T, Wahba MM. Safety models incorporating graph theory based transit indicators. Accident; analysis and prevention. 2013;50:635–44. 10.1016/j.aap.2012.06.012 22831497

[8] Quintero-Cano L. Graph theory based transit indicators applied to ridership and safety models: University of british columbia (vancouver); 2011.

[9] Menichetti G, Remondini D, Panzarasa P, Mondragón RJ, Bianconi G. Weighted Multiplex Networks. PloS one. 2014;9(6):e97857 10.1371/journal.pone.0097857 24906003 PMC4048161

[10] Sen P, Dasgupta S, Chatterjee A, Sreeram P, Mukherjee G, Manna S. Small-world properties of the Indian railway network. Physical Review E. 2003;67(3):036106 10.1103/PhysRevE.67.036106 12689131

[11] Seaton KA, Hackett LM. Stations, trains and small-world networks. Physica A: Statistical Mechanics and its Applications. 2004;339(3):635–44. 10.1016/j.physa.2004.03.019

[12] Sienkiewicz J, Hołyst JA. Statistical analysis of 22 public transport networks in Poland. Physical Review E. 2005;72(4):046127 10.1103/PhysRevE.72.046127 16383488

[13] Latora V, Marchiori M. How the science of complex networks can help developing strategies against terrorism. Chaos, solitons & fractals. 2004;20(1):69–75. 10.1016/S0960-0779(03)00429-6

[14] Latora V, Marchiori M. Efficient behavior of small-world networks. Physical review letters. 2001;87(19):198701 10.1103/PhysRevLett.87.198701 11690461

[15] Latora V, Marchiori M. Economic small-world behavior in weighted networks. The European Physical Journal B-Condensed Matter and Complex Systems. 2003;32(2):249–63.

[16] Wu J, Gao Z, Sun H. Statistical Properties of Individual Choice Behaviors on Urban Traffic Networks. Journal of Transportation Systems Engineering and Information Technology. 2008;8(2):69–74. 10.1016/s1570-6672(08)60019-7

[17] Sun HJ, Zhao H, Wu JJ. A robust matching model of capacity to defense cascading failure on complex networks. Physica A: Statistical Mechanics and its Applications. 2008;387(25):6431–5. 10.1016/j.physa.2008.07.028

[18] Wu J-J, Gao Z-Y, Sun H-j. Optimal traffic networks topology: A complex networks perspective. Physica A: Statistical Mechanics and its Applications. 2008;387(4):1025–32. 10.1016/j.physa.2007.10.014

[19] Wu J, Gao Z, Sun H. Effects of the cascading failures on scale-free traffic networks. Physica A: Statistical Mechanics and its Applications. 2007;378(2):505–11. 10.1016/j.physa.2006.12.003

[20] Wu J, Sun H, Gao Z. Cascading failures on weighted urban traffic equilibrium networks. Physica A: Statistical Mechanics and its Applications. 2007;386(1):407–13. 10.1016/j.physa.2007.08.034

[21] Wu J-j, Gao Z-y, Sun H-j. Cascade and breakdown in scale-free networks with community structure. Physical Review E. 2006;74(6):066111 10.1103/PhysRevE.74.066111 17280125

[22] Li Y, Zhou W, Guo S-j. An Analysis of Complexity of Public Transportation Network in Shanghai. Systems Engineering. 2007;1:006.

[23] Zhang J, Zhao M, Liu H, Xu X. Networked characteristics of the urban rail transit networks. Physica A: Statistical Mechanics and its Applications. 2013;392(6):1538–46. 10.1016/j.physa.2012.11.036

[24] Liu Z-q, Song R. Reliability analysis of Guangzhou rail transit with complex network theory. Journal of Transportation Systems Engineering and Information Technology. 2010;10(5):194–200.

[25] Wang Z-q, Xu R-h. Reliability Simulation Analysis of Urban Rail Transit Networks Based on Complex Network [J]. Journal of System Simulation. 2009;20:6670–4.

[26] Taylor MA, Sekhar SV, D'Este GM. Application of accessibility based methods for vulnerability analysis of strategic road networks. Networks and Spatial Economics. 2006;6(3–4):267–91. 10.1007/s11067-006-9284-9

[27] Mohamad H. Rail Transportation in Kuala Lumpur. Japan Railway and Transport Review. 2003;35:21–7.

[28] Almselati ASI, Rahmat R, Jaafar O. An overview of urban transport in Malaysia. Social Sci. 2011;6:24–33. 10.3923/sscience.2011.24.33

[29] Barter PA. Transport, urban structure and 'lock-in' in the Kuala Lumpur Metropolitan Area. International Development Planning Review. 2004;26(1):1–24. 10.3828/idpr.26.1.1

[30] Dziauddin MF, Alvanides S, Powe N. Estimating the effects of light rail transit (LRT) system on the property values in the Klang Valley, Malaysia: A hedonic house price approach. Jurnal Teknologi. 2013;61(1). 10.11113/jt.v61.1620

[31] Ore O. Hamilton connected graphs. J Math Pures Appl. 1963;42(9):21–7.

[32] Watts DJ, Strogatz SH. Collective dynamics of ‘small-world’ networks. Nature. 1998;393(6684):440–2. 10.1038/30918 9623998

[33] Seidman SB. Network structure and minimum degree. Social networks. 1983;5(3):269–87. 10.1016/0378-8733(83)90028-X

[34] Freeman LC. A set of measures of centrality based on betweenness. Sociometry. 1977:35–41. 10.2307/3033543

[35] Motter AE, Lai Y-C. Cascade-based attacks on complex networks. Physical Review E. 2002;66(6):065102 10.1103/PhysRevE.66.065102 12513335

[36] Penn A, Hillier B, Banister D, Xu J. Configurational modelling of urban movement networks. Environment and Planning B-Planning & Design. 1998;25(1):59–84. 10.1068/b250059

[37] Hillier B, Iida S. Network and psychological effects in urban movement Spatial information theory: Springer; 2005 p. 475–90.

[38] Huang W, Chow TW. Effective strategy of adding nodes and links for maximizing the traffic capacity of scale-free network. Chaos: An Interdisciplinary Journal of Nonlinear Science. 2010;20(3):033123 10.1063/1.3490745 20887063

