# Supplementary material for: Complex Network Theory Applied to the Growth of Kuala Lumpur’s Public Urban Rail Transit Network
Source: PLoS One. 2015 Oct 8;10(10):e0139961. doi: 10.1371/journal.pone.0139961 (PMC4598035; doi:10.1371/journal.pone.0139961)
Supplement: S1 Table — (PDF) [file pone.0139961.s001.pdf]

S1 Table. The list of stations

No	Station name	No.	Station name	No.	Station name	No.	Station name	No.	Station name
1	Rawang	41	Port Klang	81	Abdullah Hukum	121	Medan Tuanku	161	Subang Alam
2	Kuang	42	Sentul Timur	82	Kerinchi	122	Chow Kit	162	Putra Heights
3	Sungai Buloh	43	Sentul	83	Universiti	123	Titiwangsa	163	Sungai Buloh
4	Kepong	44	Titiwangsa	84	Taman Jaya	124	Mid Valley	164	Kampung Selamat
5	Segambut	45	PWTC	85	Asia Jaya	125	Kepong Sentral	165	Kwasa Damansara
6	Putra	46	Sultan Ismail	86	Taman Paramount	126	Rasa	166	Kwasa Sentral
7	Bank Negara	47	Bandaraya	87	Taman Bahagia	127	Batang Kali	167	Kota Damansara
8	Kuala Lumpur	48	Masjid Jamek	88	Kelana Jaya	128	Serendah	168	Surian
9	Seputeh	49	Plaza Rakyat	89	Subang Depot	129	Kuala Kubu Bharu	169	Mutiara Damansara
10	Salak Selatan	50	Hang Tuah	90	Subang Jaya	130	Tanjung Malim	170	Bandar Utama
11	Bandar Tasik Selatan	51	Pudu	91	Gombak	131	Batu Caves	171	TTDI
12	Serdang	52	Chan Sow Lin	92	Taman Melati	132	Taman Wahyu	172	Phileo Damansara
13	Kajang	53	Miharja	93	Wangsa Maju	133	Kampung Batu	173	Semantan
14	UKM	54	Maluri	94	Setiawangsa	134	Batu Kentomen	174	KL SENTRAL
15	Bangi	55	Pandan Jaya	95	Jelatek	135	Sentul	175	Muzium Negara
16	Batang Benar	56	Pandan Indah	96	Dato' Keramat	136	Sri Rampai	176	Pasar Seni
17	Nilai	57	Cempaka	97	Damai	137	Senawang	177	Merdeka
18	Labu	58	Cahaya	98	Ampang Park	138	Sungai Gadut	178	Bukit Bintang
19	Tiroi	59	Ampang	99	Kampung Baru	139	Awan Besar	179	Tun Razak Exchange
20	Seremban	60	Sentul Timur	100	Dang Wangi	140	Muhibbah	180	Cochrane
21	Putra	61	Sentul	101	Masjid Jamek	141	Alam Sutera	181	Maluri
22	Bank Negara	62	Titiwangsa	102	KL Sentral	142	Kinrara BK5	182	Taman Bukit Ria
23	Kuala Lumpur	63	PWTC	103	KL Sentral	143	Eco City	183	Taman Midah
24	Angkasapuri	64	Sultan Ismail	104	KL Sentral	144	IOI Puchong Jaya	184	Taman Mutiara
25	Pantai Dalam	65	Bandaraya	105	KL Sentral	145	Pusat Bandar Puchong	185	Taman Connaught
26	Petaling	66	Masjid Jamek	106	KLIA2	146	Taman Perindustrian Puchong	186	Taman Suntex
27	Jalan Templer	67	Plaza Rakyat	107	KL Sentral	147	Bandar Puteri	187	Sri Raya
28	Kampung Dato Harun	68	Hang Tuah	108	Bandar Tasik Selatan	148	Puchong Perdana	188	Bandar Tun Hussein Onn
29	Seri Setia	69	Pudu	109	Putrajaya/ Cyberjaya	149	Puchong Prima	189	Bukit Dukung
30	Setia Jaya	70	Chan Sow Lin	110	Salak Tinggi	150	Putra Heights	190	Taman Koperasi Cuepacs
31	Subang Jaya	71	Sungai Besi	111	KLIA	151	Lembah Subang	191	Sungai Kantan
32	Batu Tiga	72	Cheras	112	KLIA2	152	Ara Damansara	192	Bandar Kajang
33	Shah Alam	73	Salak Selatan	113	KL Sentral?	153	Glenmarie	193	Kajang
34	Padang Jawa	74	Bandar Tun Razak	114	Tun Sambanthan	154	SS15		
35	Bukit Badak	75	Bandar Tasik Selatan	115	Maharajalela	155	SS18		
36	Klang	76	Bukit Jalil	116	Hang Tuah	156	USJ 7		
37	Teluk Pulai	77	Sri Petaling	117	Imbi	157	Taipan		
38	Teluk Gadong	78	KLCC	118	Bukit Bintang	158	Wawasan		
39	Kampung Raja Uda	79	Pasar Seni	119	Raja Chulan	159	USJ 21		
40	Jalan Kastam	80	Bangsar	120	Bukit Nanas	160	Alam Megah		
